# Resting state electroencephalographic brain activity in neonates can predict age and is indicative of neurodevelopmental outcome

**DOI:** 10.1016/j.clinph.2024.05.002

**Published:** 2024-07

**Authors:** Amir Ansari, Kirubin Pillay, Emad Arasteh, Anneleen Dereymaeker, Gabriela Schmidt Mellado, Katrien Jansen, Anderson M. Winkler, Gunnar Naulaers, Aomesh Bhatt, Sabine Van Huffel, Caroline Hartley, Maarten De Vos, Rebeccah Slater, Luke Baxter

**Affiliations:** a Department of Electrical Engineering (ESAT), STADIUS Center for Dynamical Systems, Signal Processing and Data Analytics, KU Leuven, Leuven, Belgium; b Department of Paediatrics, University of Oxford, Oxford, UK; c Department of Neonatology, Wilhelmina Children's Hospital, University Medical Center Utrecht, Utrecht, Netherlands; d Department of Development and Regeneration, University Hospitals Leuven, Neonatal Intensive Care Unit, KU Leuven, Leuven, Belgium; e Department of Development and Regeneration, University Hospitals Leuven, Child Neurology, KU Leuven, Leuven, Belgium; f Department of Human Genetics, University of Texas Rio Grande Valley, Brownsville, TX, USA

**Keywords:** Infant, Deep learning, Convolutional neural network, Electroencephalography, Bayley Scale, Brain age gap

## Abstract

**Objective:**

Electroencephalography (EEG) can be used to estimate neonates’ biological brain age. Discrepancies between postmenstrual age and brain age, termed the brain age gap, can potentially quantify maturational deviation. Existing brain age EEG models are not well suited to clinical cot-side use for estimating neonates’ brain age gap due to their dependency on relatively large data and pre-processing requirements.

**Methods:**

We trained a deep learning model on resting state EEG data from preterm neonates with normal neurodevelopmental Bayley Scale of Infant and Toddler Development (BSID) outcomes, using substantially reduced data requirements. We subsequently tested this model in two independent datasets from two clinical sites.

**Results:**

In both test datasets, using only 20 min of resting-state EEG activity from a single channel, the model generated accurate age predictions: mean absolute error = 1.03 weeks (p-value = 0.0001) and 0.98 weeks (p-value = 0.0001). In one test dataset, where 9-month follow-up BSID outcomes were available, the average neonatal brain age gap in the severe abnormal outcome group was significantly larger than that of the normal outcome group: difference in mean brain age gap = 0.50 weeks (p-value = 0.04).

**Conclusions:**

These findings demonstrate that the deep learning model generalises to independent datasets from two clinical sites, and that the model’s brain age gap magnitudes differ between neonates with normal and severe abnormal follow-up neurodevelopmental outcomes.

**Significance:**

The magnitude of neonates’ brain age gap, estimated using only 20 min of resting state EEG data from a single channel, can encode information of clinical neurodevelopmental value.

## Highlights

• Accurate infant age predictions can be made using 20 min resting state EEG from a single channel.
• The deep learning age prediction model generalises to two independent datasets from two different clinical sites.
• The magnitude of the brain age gap differs between infant groups with different Bayley Scale outcomes.

## 1 Introduction

The newborn infant’s brain is undergoing rapid developmental change, influenced by both genetic and environmental factors (Colonnese et al., 2010, Milh et al., 2007, Wess et al., 2017). Relative to their term-born counterparts, infants born prematurely are at increased risk of poorer long-term neurodevelopmental outcomes (Blencowe et al., 2013, Wallois et al., 2020). This risk of impairment increases with the degree of prematurity at birth and the presence of gross morphological lesions but can also be brought about by subtler environmental stressors (Scher, 2008), excessive exposure to painful stimuli (Grunau, 2013, Moultrie et al., 2017), and pharmacological interventions (Duerden et al., 2016, Malk et al., 2014).

The early identification of abnormal neurodevelopment is essential to identify infants at greatest risk who might benefit most from developmental care interventions (Burke, 2018). To date, neurological assessment of the newborn has remained predominantly subjective (Dempsey et al., 2018). For example, trained neonatologists and clinical neurophysiologists visually inspect infants’ brain activity using electroencephalography (EEG) to determine whether brain function is developmentally age-appropriate or dysmature (Scher, 1997) based on developmentally changing EEG features characteristic of maturational status (André et al., 2010). While these trained individuals can estimate age with an error of two weeks for preterm babies and one week for term babies, these estimates can be highly variable across reviewers (Stevenson et al., 2020b). Subjectivity, inter-rater variability, and the requirement of specialist EEG interpretation are central issues that severely limit the reliability and generalisability of many current neurological assessment methods. There is an urgent need for objective and automated neuromonitoring that can be used cot-side to identify infants at increased risk of abnormal neurodevelopmental outcomes.

To this end, a variety of metrics have been developed to capture key maturational characteristics from preterm EEG (De Wel et al., 2017, Dereymaeker et al., 2016, Lavanga et al., 2017, Pillay et al., 2018, Tolonen et al., 2007), and these measures have been combined using machine learning algorithms to successfully predict infants’ brain age (O’Toole et al., 2016, Stevenson et al., 2017). An infant’s brain age is the biological age of their brain, which is influenced by a wide array of genetic and environmental exposures (Salih et al., 2023). An infant’s postmenstrual, gestational, or chronological age (Engle et al., 2004) are not always a perfect measure of biological age, as they do not account for individual differences in rates of maturation due to differences in genetic and environmental influences (Salih et al., 2023). Models that use brain-based features (structural or functional) as predictors and postmenstrual age (PMA) as the output can be used to derive infants’ brain age. The difference between a person’s chronological (or postmenstrual) age and brain age, termed the brain age gap, has been demonstrated to be more than random noise prediction error but is of biological and clinical value in both adults (Salih et al., 2023, Smith et al., 2019, Vidal-Pineiro et al., 2021) and infants (Pillay et al., 2020, Stevenson et al., 2020a).

In infants, the magnitude of the brain age gap has been demonstrated to correlate with neurodevelopmental outcomes (Pillay et al., 2020, Stevenson et al., 2020a). These studies established the proof-of-concept in infant populations that the inter-individual variability in automatically and objectively generated brain age gaps could be used to risk-stratify infants in the first few weeks of postnatal life according to neurodevelopmental outcomes. However, a limitation to these studies is that the models needed multiple EEG channels and at least one hour of EEG recording duration. These data-heavy requirements limit the ease with which these methods can be incorporated into the busy clinical environment.

Here, we directly address these barriers to clinical ease of use. Moreover, we adopt a deep learning approach that does not require the pre-specification of features. In the current study, we implement a convolutional neural network (CNN)-based architecture to generate infant brain age predictions using reduced EEG data requirements compared to previous proof-of-concept studies. We first compare the performance of the model with varying electrode montages and recording durations in a training set and establish the fully trained model. We next validated this trained model in two independent samples, one of which was collected at a different site by an independent research team with a different recording set-up. Finally, we compared the brain age gaps for infants who had normal and abnormal neurodevelopmental outcomes assessed using the Bayley Scale of Infant and Toddler Development Second Edition (BSID-II) at 9 months of age.

## 2 Methods

### 2.1 Participants

#### 2.1.1 Study design

Data were analysed in three independent samples. The first sample, referred to as dataset 1, was used to train the model. The second and third samples, referred to as datasets 2 and 3, were used to test the trained model’s age prediction accuracy. Due to the existence of 9-month BSID-II follow-up outcomes for dataset 2, this dataset was also used to assess the model’s prediction error magnitude as a brain age gap estimate by comparing mean prediction errors among the three BSID-II outcome groups.

#### 2.1.2 Recruitment

EEG data for datasets 1 and 2 were recorded from the Neonatal Intensive Care Unit at UZ Leuven Hospitals, Leuven, Belgium. Infants were recruited, and data were recorded with informed consent from the parents and in accordance with the guidelines approved by the ethics committee of the University Hospitals, Leuven. All infants had a gestational age at birth less than 32 weeks, and between one and five recordings were obtained during their stay in the Neonatal Intensive Care Unit. Infants in dataset 3 were selected from a database of previously recorded data collected at the Newborn Care Unit and Maternity wards of the John Radcliffe Hospital, Oxford University Hospitals NHS Foundation Trust, Oxford, United Kingdom. Ethical approval was obtained from the UK National Research Ethics Service (reference: 12/SC/0447), and parental written informed consent was obtained before each participant was studied. All participant recruitment was conducted in accordance with the standards set by the Declaration of Helsinki and Good Clinical Practice guidelines.

#### 2.1.3 Datasets

A summary of the participant demographics and clinical information is presented in Table 1, grouped according to dataset. For datasets 1 and 2, which included BSID-II follow-up outcomes, infants were categorized into three groups based on their BSID-II outcomes: normal (i.e., no neurodevelopmental impairment), mild abnormal (mild neurodevelopmental impairment), and severe abnormal outcomes (mild-to-severe neurodevelopmental impairment). Normal outcomes were defined as infants with a BSID-II Mental Development Index and Psychomotor Development Index both ≥85 (Stevenson et al., 2017), absence of any severe brain lesions (from cerebral ultrasound recordings), no periventricular leukomalacia, and no use of any sedative or anti-epileptic medication during EEG recording. Mild abnormal outcomes were defined as a minimum Mental Development Index or Psychomotor Development >70 and <85. Severe abnormal outcomes had a minimum Mental Development Index or Psychomotor Development ≤70 or presence of cerebral palsy. Patients who died (i.e. passed away before 9 months follow up) were also included in this group.

**Table 1:** **Participant demographics.** Data are presented as count (percent) or mean (standard deviation). Abbreviations: BSID-II = Bayley scale of infant and toddler development, second edition; GA = gestational age; PMA = postmenstrual age.

	Dataset 1	Dataset 2	Dataset 3
Purpose	Model training	Model testing (PMA prediction; brain age gap vs BSID-II outcome relationship)	Model testing (PMA prediction)
Site	Leuven (Belgium)	Leuven (Belgium)	Oxford (UK)
Number of subjects	40	43	57
Number of recordings	111	148	73
Number of recordings per subject	2.8 (1.6)	3.4 (1.4)	1.3 (0.7)
Recording duration (hours)	8.1 (5.9)	7.1 (5.7)	0.8 (0.3)
GA at birth (weeks)	31.1 (4.9)	27.8 (4.5)	32.7 (4.7)
PMA at study (weeks)	34.6 (3.2)	32.5 (2.0)	35.2 (3.0)
Sex			
Males	13 (32.5%)	33 (76.7%)	31 (54%)
Females	27 (67.5%)	10 (23.3%)	26 (46%)
BSID-II categorisation			Unavailable
Normal	40	22	n/a
Mild abnormal	0	11	n/a
Severe abnormal	0	10	n/a
Patent ductus arteriosus	8 (20%)	7 (16.3%)	8 (14%)
Necrotizing enterocolitis	2 (5%)	1 (2.3%)	5 (9%)
Previous infection (with antibiotic treatment)	15 (37.5%)	22 (51.2%)	30 (53%)
Mean duration on mechanical ventilation (days)	16.2 (19.4)	17.6 (20.6)	2.1 (8.4)

Dataset 1 consisted of *n* = 40 infants (111 recordings) with a PMA range at the time of recording of 27.3–43.1 weeks, with a mean recording duration of 8 h 07 m (standard deviation: 5 h 55 m) and a mean number of recordings per infant of 2.8 (standard deviation: 1.6). All infants in dataset 1 were selected for normal neurodevelopmental outcome at 24-month follow-up age based on behavioural assessment using BSID-II.

Dataset 2 consisted of *n* = 43 infants (148 recordings) with a PMA range at the time of recording of 27.3–42.0 weeks, a mean recording duration of 7 h 05 m (standard deviation: 5 h 43 m), and a mean number of recordings per infant of 3.4 (standard deviation: 1.4). This dataset includes infants with a range of both normal and abnormal 9-month follow-up BSID-II outcomes. *N* = 22 infants (73 recordings) had normal outcomes; *n* = 11 infants (37 recordings) had mild abnormal outcomes; and *n* = 10 infants (38 recordings) had moderate-to-severe abnormal outcomes (Pascal et al., 2020).

Dataset 3 consisted of *n* = 57 infants (73 recordings) with a PMA range at the time of recording of 28–42.6 weeks, with a mean recording duration of 50 min (standard deviation: 18 min) and a mean number of recordings per infant of 1.3 (standard deviation: 0.7). Infants were included in this dataset for the current study if they had at least 20 min of EEG data recorded and if the EEG was assessed as normal for age by a trained clinical neurophysiologist (author GSM).

### 2.2 EEG data

#### 2.2.1 Setup

For datasets 1 and 2, data were recorded using a sampling frequency of 250 Hz using Brain RT OSG Equipment (Mechelen, Belgium). In a few cases, the EEG was sampled at 256 Hz due to some setup variations on the Brain RT device used. All recordings were performed with nine electrodes in a referential montage: Fp1, Fp2, C3, C4, T3, T4, O1, O2, and Cz reference.

For dataset 3, EEG recordings were acquired from DC to 800 Hz using a SynAmps RT 64-channel headbox and amplifiers (Compumedics Neuroscan). Activity was recorded using the CURRY scan7 neuroimaging suite (Compumedics Neuroscan), with a sampling rate of 2000 Hz. Between 8 and 25 electrodes were used for recording, positioned according to the modified international 10–20 system, including C3 and C4 (those used in the analysis here), with reference at Fz and ground at Fpz. The scalp was cleaned with preparation gel (Nuprep gel, D.O. Weaver and Co.), and disposable Ag/AgCl cup electrodes (Ambu Neuroline) were placed with conductive paste (Elefix EEG paste, Nihon Kohden).

For all datasets, EEG recordings were conducted in the infant’s cot or incubator on the neonatal units. Measurements were taken at different times of the day, but in all cases, monitoring occurred during periods when the neonates were relaxed and typically asleep, to not unnecessarily stress the baby.

#### 2.2.2 Preprocessing

For dataset 1, each recording was downsampled to 64 Hz, which included an anti-aliasing filter. Recordings were then split into 30-second segments, and the amplitudes were standardized such that the mean and standard deviation of the amplitudes were zero and one, respectively. The mean and standard deviation were obtained by standardizing the data across all channels. Finally, any segments where the absolute differences (compared to the mean) at any point exceeded 600 µV were rejected as artefacts. For datasets 2 and 3, pre-processing was matched to dataset 1. For the standardization of datasets 2 and 3, the mean and standard deviation of dataset 1 were used.

### 2.3 Training the age prediction model in dataset 1

#### 2.3.1 Model architecture

**Fig. 1:**
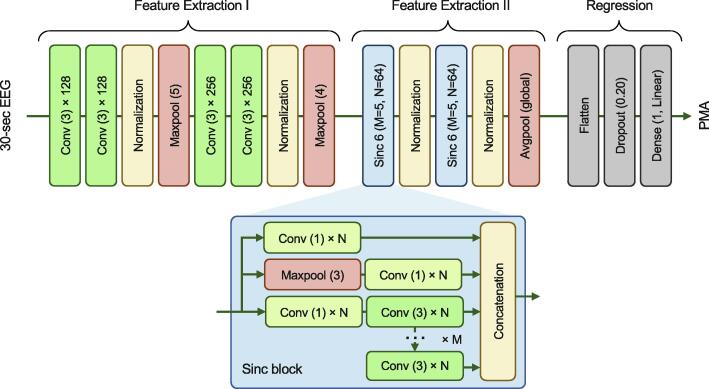
**Block diagram of the deep learning model architecture.** The neural network architecture is separated into three blocks: Feature Extraction I, Feature Extraction II, and Regression. During Feature Extraction 1, a recognised CNN structure is used, consisting of multiple Conv layers to extract features from the EEG, as well as normalization layers for stable training and Maxpool layers to aggregate these features and provide some local temporal invariance of the features. This block effectively extracts and separates out the main characteristics of the EEG signal. In Feature Extraction II, a similar structure is used but replacing the single Conv layer with the Sinc block as a layer. As shown, a single Sinc layer consists of a set of Conv layers that effectively extract features from the EEG at varying scales and combines them in an efficient way via parameter sharing. With this Feature Extraction II block, the now-separated features from Feature Extraction I are further processed across different temporal scales to generate more fine-tuned features from the original EEG. The final regression block provides a Linear layer that regresses the generated feature to a PMA estimate. The Flatten and Dropout layers here restructure the feature output to facilitate this and assists in improving model training. Abbreviations: Avgpool = average pooling layer; CNN = convolutional neural network; Conv = convolutional layer; EEG = electroencephalography; Maxpool = maximum pooling layer; PMA = postmenstrual age; Sinc = shared inception block.

Fig. 1 shows the block diagram of the deep neural network for brain age prediction. As input, the network processes a 30 s multichannel EEG segment. Each input segment has dimensions C x 1920, where C is the number of EEG channels and 1920 is the total number of timepoints in the 30 s segment (30 s duration × 64 Hz sampling frequency). Each segment has a single output label that is a continuous PMA value.

The model includes a series of convolutional layers with exponential linear unit activations, maximum and average pooling layers to downsample the data, normalization layers for faster training convergence, and a dense layer with linear activation to perform the final regression and produce a brain age estimate. As each convolutional layer is designed to extract specific characteristics from the EEG, these are analogous to a (trainable, data-driven) feature extraction layer. More generally, the proposed architecture can be grouped into a more traditional, sequential CNN block that can be described as an initial feature extraction stage, followed by the two successive Sinc (i.e., shared inception) blocks that form a second feature extraction stage (Ansari et al., 2021).

#### 2.3.2 Model training

Dataset 1 was divided by recording into training and test sets of size 64 and 47 recordings, respectively. These were age-stratified by first dividing PMA into two-week intervals (27–28, 29–30, …, 42–43 weeks PMA). Each recording within an interval was then randomly assigned to either the training or test set with 50% probability, ensuring a good representation in both sets across PMA. A recording-wise test-train split was chosen because it allowed a better stratification by age than splitting by infant. While a recording-wise split does not guarantee full statistical independence between the training and test sets of dataset 1 due to recordings from a single infant possibly featuring in both sets, final model performance assessment is only considered for datasets 2 and 3, which have no information leakage from the training set (dataset 1).

To prevent over-fitting during model training, early stopping was used by assessing the change in model performance based on a validation set. The validation set was formed by removing the last 25% of each recording in the training set. This ensured that the validation set was stratified in the same way as the training set such that model updates during training were always based upon a good age representation in the data.

Two sources of Gaussian noise were added to the deep learning networks to improve robustness. First, Gaussian noise (standard deviation = 0.001) was added to the standardized input EEG. This can help the network overcome noisy EEG and is a common approach used to prevent overfitting in deep learning models to noise in the data and has shown success across many other deep learning applications (Audhkhasi et al., 2013, Bishop, 1995, Ghose et al., 2020, Koistinen and Holmstrom, 1991, Vincent et al., 2010, Yin et al., 2015). Injecting small random noise to the input signal helps the network learn to ignore such noisy patterns and therefore better generalise to new, unseen datasets. Additionally, Gaussian noise (standard deviation = 1 day) was added to the PMA target labels. As the PMAs of the recordings are sparsely scattered (and repeated for each segment in a recording), this helped the network tolerate small prediction errors and further improved the generalization performance. The added Gaussian noise, with a deviation of 1 day, is small relative to the inherent uncertainty of an infant’s PMA determined clinically, which, according to the American Academy of Paediatrics, can vary by as much as two weeks (Engle et al., 2004).

The EEG recording was fully segmented into contiguous 30 s segments (e.g., a 1 hr recording was segmented into 120 segments). As the durations of the recordings are not consistent, conventional segmentation into 30 s segments using a sliding window ensures that longer duration recordings are more emphasised during training, resulting in a bias. To solve this, a fixed number of segments (*n* = 1000) is picked at random from every recording with replacement (bootstrapping) for each batch during training. This results in a total of 6.5 M bootstrapped segments per training epoch.

The model produces a brain age prediction for each 30 s recording segment, and the set of estimates per recording is aggregated into a single predicted brain age value. To partially correct for the training bias resulting from the non-uniform distribution of the data with PMA, a training weight is assigned to each segment depending on the frequency of their corresponding PMA. To calculate these training weights (or class weights), an approach used in classification tasks was employed by grouping the PMAs into ranges and calculating each weight according to the following formula: {weight for class i} = {number of samples}/({number of classes} * {number of samples in class i}) (King and Zeng, 2001). Consequently, segments from recordings with more common PMAs have less impact on each network update during training.

Finally, as neural network training is a non-convex problem and requires a stochastic initialisation of the parameters, each trained network is not unique. Consequently, the final performance of these trained networks varies. To achieve a robust solution, a deep ensemble approach was used by repeatedly training the model ten times using different random initialisations i.e., a 10-learner ensemble method (Fort et al., 2020).

#### 2.3.3 Model assessment

The ultimate goal of the prediction model is to generate a single brain age prediction estimate per EEG recording. The model generates ten brain age prediction estimates per 30 s segment of an EEG recording (as a 10-learner ensemble method was used). During testing, all contiguous 30 s segments across each recording are used with the number of 30 s segments therefore dependent on the overall EEG recording duration. To aggregate a deep learning model’s predictions to a single value per recording, the median across the ten ensemble predictions per 30 s segment is determined, and then a further median across all 30 s segments in the recording is taken, resulting in the final prediction estimate. Across all recordings in the test set in dataset 1, there were a total of 30 K segments used. The final prediction estimate for a recording is used to generate the prediction error (or absolute prediction error) for that recording.

Deep neural networks are notorious for being black-box machines, limiting interpretability when compared to machine learning approaches and traditional visual assessment approaches. To help understand the model’s functioning, we used a method that we refer to as input-loss minimisation in this paper. When a neural network model is trained, the weights are adapted by backpropagating the loss derivatives through the network as labelled data is added to the model in batches during training via versions of stochastic gradient descent. In input-loss minimization, however, we now freeze the trained model (i.e. the weights are now fixed) and specify a ‘target’ PMA as the output. Input ‘EEG’ is provided as Gaussian noise and the backwards and forwards propagation (still using stochastic gradient descent) is allowed to commence but this time with the derivatives of the loss with respect to the input and target PMA allowed to change instead of the (now fixed and trained) weights. The result is that the input begins to be modified to reflect synthetic EEG that the model assumes represents the target. Inspired by the activation maximization visualization method (Erhan et al., 2009), changes to the input in this way as optimized by the neural network may reveal potentially important physiological patterns that the network has identified to estimate the target PMA. Using this method, we generated synthetic EEG data for three target postmenstrual weeks: PMA = 30, 35, and 40 weeks. These synthetic EEG outputs are qualitatively assessed based on known EEG maturational features over this age range (André et al., 2010) to facilitate interpretation of the Sinc model’s functioning.

#### 2.3.4 Reducing EEG channels

**Fig. 2:**
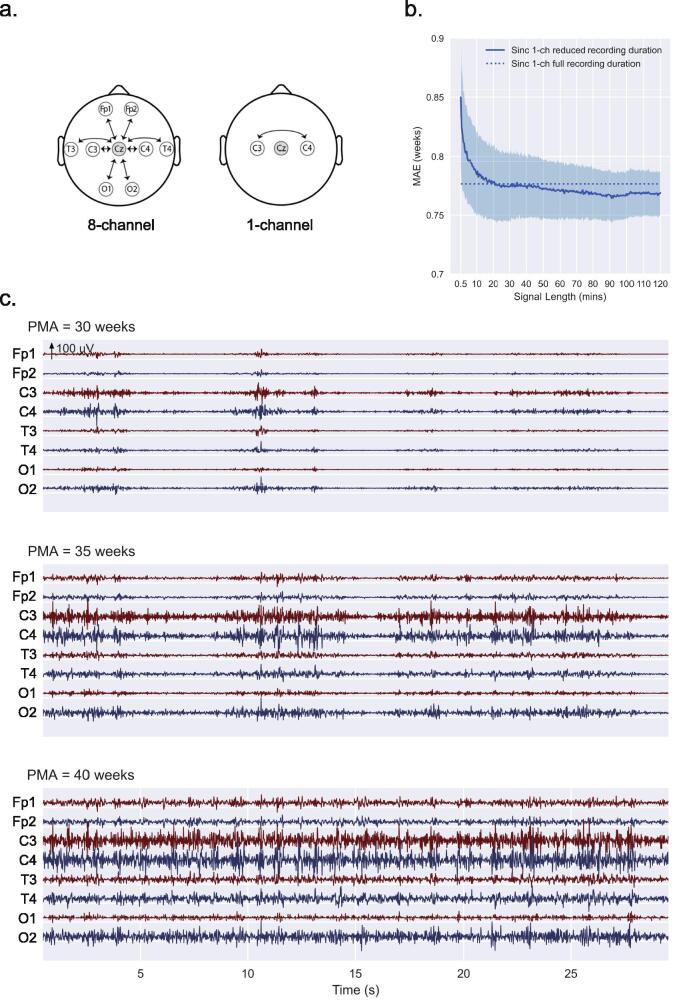
**Training the age prediction model in dataset 1.** (a) Reducing channel number. EEG montages used during analysis, with the reference electrode Cz shaded in grey. Arrows represent the specific channels used during analysis. During model training in dataset 1, both the 8-channel referential montage and 1-channel bipolar montage were used. For both datasets 2 and 3, only the 1-channel bipolar montage was used. (b) Reducing recording duration. Assessment of age prediction error (MAE on y-axis) in dataset 1 as EEG recording duration is varied from 0.5-120 min (x-axis), benchmarked using the full recording duration. The full recording duration MAE (0.78 weeks) is displayed as the horizontal dotted line. The MAEs for reduced recording durations are displayed as the mean (solid blue line) ± standard deviation error bars (shaded blue). Performance using the reduced recording durations is matched to the full recording duration when recordings of 20 min or longer are used; using a recording duration of less than 20 min exhibits a gradual drop in prediction performance (larger MAE values). Note, the performance of the reduced recording durations of 45–120 mins appear to outperform the full recording duration; however, this difference in MAE is relatively minor and is not consistent beyond 120mins suggesting a trivial noise or bias effect, that will be limited to this training dataset. (c) Synthetic EEG data generated using the deep learning model. These simulated EEG data highlight changes in discontinuity characteristics with PMA, reminiscent of maturational trends seen in real EEG data. The results are generated using the input-loss minimization technique for three target PMAs (30, 35, and 40 weeks) spanning the early preterm to term age range using the model trained on the 8-channel full recording duration EEG dataset 1. The degree of continuity in activity can be seen to increase with PMA. Abbreviations: EEG = electroencephalography; MAE = mean absolute error; PMA = postmenstrual age; Sinc = shared inception block. (For interpretation of the references to colour in this figure legend, the reader is referred to the web version of this article.)

The deep learning model was initially trained using an 8-channel referential montage setup and the full recording duration. Subsequently, the model was re-trained, and performance was assessed by changing the EEG montage to a 1-channel bipolar (C3-C4) montage (Fig. 2a). The 1-channel bipolar montage was selected for its similarity to setups used in clinical amplitude-integrated EEG monitors. EEG pre-processing was independently repeated, with the amplitude standardisation step recalculated on the reduced channel configuration. It is worth noting that the 1-channel bipolar montage used for our analyses was achieved by ignoring the additional channels unnecessary for this montage. This approach is distinct from a true clinical scenario when only a 1-channel bipolar montage would be used during recording. Our assumption, which we believe to be reasonable, is that both approaches to the 1-channel bipolar montage setup are closely matched for this specific use case. However, this assumption should be tested in future external validations of the deep learning model using clinical grade bipolar montage data.

#### 2.3.5 Reducing EEG recording duration

Having demonstrated the reasonable performance of the model using the full-length EEG recording duration with a 1-channel bipolar setup, we next assessed the model performance using the 1-channel bipolar setup as the EEG recording duration was systematically varied over a range of recording lengths from 0.5–120 min. To obtain a reduced recording from a single full recording, we randomly sampled each reduced duration segment from the full recording, generating an absolute error value per reduced duration segment. Due to the arbitrary nature of selecting a reduced recording segment from a full recording, we repeated the procedure using 1000 bootstrapped samples from which a mean absolute error was derived per recording per reduced recording duration. A minimum reduced recording duration was identified as the duration at which the prediction performance, measured using the mean absolute error, noticeably drops below that of the full duration model.

### 2.4 Predicting age in independent datasets 2 and 3

The model was trained using only dataset 1 with the 1-channel bipolar setup and was not adapted or re-trained when applied to the independent datasets 2 and 3. When applying the model, both datasets 2 and 3 used the 1-channel bipolar montage (C3-C4) (Fig. 2a). For dataset 2, the 20 min recording duration was randomly sampled from the full duration EEG recording; for dataset 3, due to the much shorter recording durations, the first 20 min of each recording were used.

For both datasets 2 and 3, the model performance was assessed by calculating the mean absolute error (MAE), with 95% confidence intervals (CIs) estimated using bootstrapping: bias corrected and accelerated percentile (BCA) method with 10,000 bootstrap samples (MATLAB R2023a). One-tailed significance testing, with a 5% significance level, was performed using permutation testing via FSL’s PALM: Freedman-Lane method with 10,000 permutations (Winkler et al., 2014). Due to multiple recordings per infant existing in both datasets, permutations were limited to appropriate exchangeability blocks, and the hierarchical data structure is visualised in the supplementary information using tree diagrams as per the original methods paper (Winkler et al., 2015). Last, in addition to the MAE (an absolute measure of performance in original units of weeks), we report the coefficient of determination (R^2^) as a complementary relative measure of performance, computed using the sum-of-squares formulation, which indicates the proportion of variance explained (Poldrack et al., 2019).

Due to the presence of a small number of extreme values, we performed sensitivity tests using robust measures of performance, median absolute error and robust R^2^, as these alternative versions of the performance metrics are insensitive to outliers (Kvalseth, 1985, Poldrack et al., 2019).

### 2.5 Assessing the potential value of the prediction error magnitude in dataset 2

Using dataset 2, the association between infants’ brain age prediction error and their 9-month BSID-II follow-up outcomes was assessed to test the potential value of the model’s prediction error magnitude as an estimate of a meaningful biological brain age gap. A brain age gap was determined per recording using a multistep procedure: the signed difference between PMA and predicted PMA was derived, and then PMA was regressed on these signed differences to generate signed difference residuals that no longer had a PMA linear association (Le et al., 2018, Smith et al., 2019). The absolute value of these residualised errors was then used as the estimate of brain age gap magnitude per recording. Finally, to obtain a single brain age gap magnitude per subject, the mean brain age gap magnitude was taken across all recordings per subject. Due to the presence of a small number of extreme values, we also performed a sensitivity test by taking the median brain age gap across an infant’s recordings.

Subjects were grouped according to their 9-month BSID-II: normal, mild abnormal, and severe abnormal. The brain age gap magnitudes are displayed for visualisation using Cumming estimation plots, implemented using the dabestr (Data Analysis using Bootstrap-Coupled ESTimation) package in R, with which 95% CIs are generated using the BCA method with 5000 samples (Ho et al., 2019).

To test significant differences in the mean MAE among the three groups, all three pairwise two-sided t-tests were performed. All tests were adjusted for the number of recordings per subject to account for potentially different signal-to-noise ratios due to differing numbers of averaged recordings among subjects. The 5% familywise error rate was controlled to account for multiple comparisons using the non-parametric permutation-based Westfall–Young method, as implemented in PALM (Alberton et al., 2020).

## 3 Results

### 3.1 Training the age prediction model using reduced EEG requirements

Using dataset 1, the model was initially trained using all eight channels and full recording durations, resulting in an MAE = 0.73 weeks. We next reduced the number of EEG channels from the 8-channel referential montage to a 1-channel bipolar montage (Fig. 2a) and retrained the model using full recording durations. This model resulted in an MAE = 0.78 weeks. Finally, we used the 1-channel bipolar montage to retrain the model on progressively shorter recording durations, ranging from 0.5 to 120 mins. Using only 20 min of EEG recording, the model prediction error was approximately equivalent to using the full recording duration (Fig. 2b). The final model was trained on dataset 1 using the 1-channel bipolar montage and 20 min recording duration (MAE = 0.79 weeks).

To shed light on the EEG features that might be driving the age predictions, we generated synthetic EEG data from the trained model using input-loss minimisation. This was performed using the 8-channel, full recording duration data to achieve a reasonable signal-to-noise ratio. Visually inspecting the synthetic data for 30, 35, and 40 weeks PMA, the signal continuity and duration of bursts increased with increasing PMA (Fig. 2c). The 30-week synthetic data reflect aspects of high discontinuity with short, high amplitude bursts and long-duration inter-burst intervals. With increasing PMA, the inter-burst interval durations decreased and burst periods widened, and by term age, the signal was almost fully continuous with no clear burst or inter-burst interval patterns. These observations may suggest that the 1-channel 20 min recording duration model may also use similar EEG characteristics, such as signal continuity and bursting, to predict infant age.

### 3.2 Age is accurately predicted in two independent datasets

**Fig. 3:**
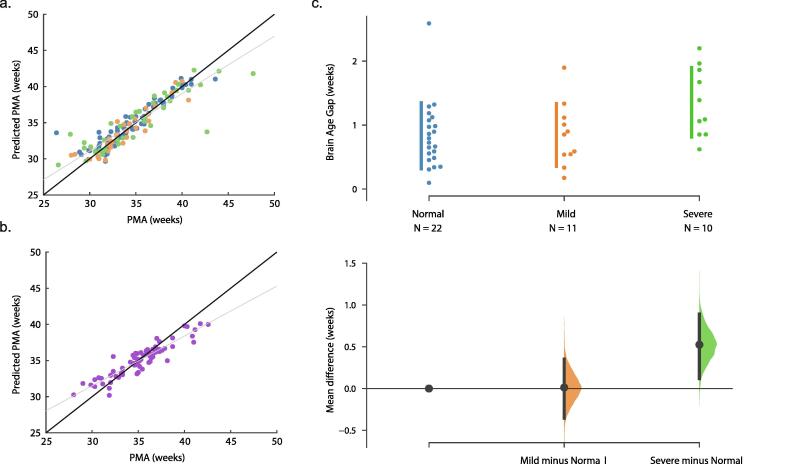
**Model performance assessed in two independent datasets from two clinical sites.** (a) and (b) display the age prediction accuracy results, with PMA on the x-axis and predicted PMA on the y-axis for each recording per dataset, where each dot represents one recording. The black y = x line is the line of perfect prediction. The grey line is the least squares fit line. In (a), infants are grouped according to their 9-month BSID-II follow-up outcomes: normal is blue, mild abnormal is orange, and severe abnormal is green. In (b), no follow-up outcomes were available. (c) The brain age gap results are displayed using a Cumming estimation plot. Top: In the swarm plots, each dot represents one subject, and subjects are grouped according to their BSID-II follow-up outcomes (same colour coding as (a)). The y-axis is the brain age gap magnitude: absolute value of prediction error with PMA association bias removed (residualised). Next to each swarm plot is a vertical line which is the ± standard deviation error bar. Bottom: The normal outcome group is used as a common control for the mild and severe abnormal groups. The solid circles represent the group mean minus the mean of the shared control, and the vertical black bars are the 95% confidence interval, determined using bootstrap resampling. The resampled distribution is also displayed. The severe outcome group had a significantly larger mean brain age gap (p-value = 0.04), assessed using two-sided t-tests, controlled for number of recordings per subject, and p-values adjusted for multiple comparisons. Abbreviations: PMA = postmenstrual age. (For interpretation of the references to colour in this figure legend, the reader is referred to the web version of this article.)

We applied the trained model to two independent datasets. First, the model was applied to a cohort of infant data (dataset 2) collected at the same site as the training data. Using 1-channel bipolar EEG data of 20 min recording duration, the model was able to accurately predict infant age (Fig. 3a): *n* = 43 subjects (148 recordings), R^2^ = 0.82, MAE = 1.03 weeks, 95% CI = [0.87, 1.28], *p* = 0.0001. The exchangeability block structure used in the permutation test to account for multiple recordings per infant is depicted in Fig. S1a.

Next, the model was tested on data collected at an independent site by a separate research group (dataset 3). In this second independent dataset, the model was also able to accurately predict infant age (Fig. 3b): *n* = 57 subjects (73 recordings), R^2^ = 0.81, MAE = 0.98 weeks, 95% CI = [0.80, 1.19], *p* = 0.0001. The exchangeability block structure for this dataset is depicted in Fig. S1b.

The sensitivity tests, which used median absolute error and robust R^2^, produced results in line with our primary analysis, indicating the robustness of our results to the influence of extreme values (Supplementary information S2.1).

### 3.3 The infant brain age gap contains clinically valuable prognostic information

We stratified the infants in dataset 2 based on their 9-month BSID-II outcomes: normal, mild abnormal, and severe abnormal. After correcting the prediction errors for PMA-association bias, the infants with normal BSID-II outcomes had a mean brain age gap = 0.83 weeks (*n* = 22 infants), those with mild abnormal outcomes had a mean brain age gap = 0.84 weeks (*n* = 11 infants), and those with severe abnormal outcomes had a mean brain age gap = 1.36 weeks (*n* = 10 infants). These three groups are displayed using a Cumming estimation plot with the normal group as the shared control (Fig. 3c).

We performed pairwise comparisons of the mean brain age gaps among the three groups, adjusting for the number of recordings per subject and correcting p-values for multiple comparisons using a permutation testing approach. Among the three groups (*n* = 43 subjects), the severe abnormal BSID-II outcome group had a significantly larger mean brain age gap than the normal BSID-II outcome group: difference in mean brain age gap = 0.50 weeks, t-statistic = 2.52, *p* = 0.04, Cohen’s D = 1.08. The other comparisons were non-significant. Severe vs mild BSID-II outcome group: difference in mean brain age gap = 0.49 weeks, t-statistic = 2.17, *p* = 0.09, Cohen’s D = 1.05. Mild vs normal BSID-II outcome group: difference in mean brain age gap = 0.01 weeks, t-statistic = 0.04, *p* = 1, Cohen’s D = 0.02. The sensitivity tests, which used the median age gap across recordings per subject, produced results in line with our primary analysis, indicating the robustness of our results to the influence of extreme values (Supplementary information S2.2).

## 4 Discussion

This study presents a deep learning model that predicts infants’ age from resting-state EEG activity. The model was trained using 20-minute EEG recordings from a 1-channel bipolar montage, without the need to pre-specify predictive features. The trained model was subsequently applied to two independent datasets from two clinical sites (Belgium and UK). In both test sets, the model accurately predicted infants’ age, accounting for over 80% of the age variance, which is generally considered a very large effect size (Cohen, 1992). In absolute terms, in both datasets, the prediction error (MAE) is approximately one week, which is on par with trained human assessors (Stevenson et al., 2020b) and similar accuracy to a random forest model with larger EEG data requirements (Pillay et al., 2020). Additionally, in one of the test sets that had 9-month follow-up BSID-II outcomes, the infants were stratified into normal, mild abnormal, and severe abnormal outcome groups based on their BSID-II outcomes. The model-generated brain age gaps differed among these three groups, with significantly larger brain age gaps observed in the severe outcome group than in the normal outcome group. Again, the effect size was large (Cohen’s D > 1) (Cohen, 1992). These results indicate that the age prediction model also encodes clinically relevant prognostic information in the magnitude of the brain age gaps, which could allow early identification of high-risk infants during the neonatal period.

Using the trained model to generate synthetic EEG data, our results suggest that the model’s predictive performance may rely on identifying signal characteristics related to changes in the EEG discontinuity (bursts and inter-burst intervals) with age. The progression of burst/inter-burst activity to continuous activity is the expected characteristic developmental trajectory from preterm to term age (André et al., 2010). Interestingly, these discontinuity patterns are also key for human experts when performing visual age prediction (Dereymaeker et al., 2017, Husain, 2005). Observing this link between the synthetic EEG and expected maturational trends suggests that the model may rely on biophysiologically sensible signal features.

The model’s performance did not drop substantially from eight channels to one. This might suggest that the feature extraction stages of the architecture may be more tuned to global channel-independent characteristics, such as bursting and continuity, as opposed to spatially-dependent characteristics, such as inter-channel synchrony. Furthermore, if the model relies on identifying changes in burst/inter-burst cycling and encodes this in a highly multi-scale manner, this may indicate that information on an infant’s burst/inter-burst cycling may be sufficiently discernible from a 20-minute EEG recording, with additional data providing diminished returns in discriminatory power.

The ultimate interest in studying brain age gap magnitude is that neurological dysfunction can manifest in infants’ EEG as both accelerated or slowed maturation relative to a normative trajectory (Scher, 1997, Watanabe et al., 1999), and these functional maturational deviations have prognostic value (Iyer et al., 2015, Tokariev et al., 2019). In work published by an independent group (Stevenson et al., 2020a), brain age gaps exhibited the greatest separation between infants with normal and severely abnormal BSID-II follow-up outcomes – an observation that is consistent with the current study’s findings, further supporting the results of our model.

The present study focused on the prognostic value of preterm and term age resting-state brain function as a basis for risk stratification using 9-month BSID-II follow-up as the relevant outcome. As with any scale, there are limitations to BSID-II predictive validity (Hack et al., 2005). Clinical decision making regarding the provision of developmental care interventions (Burke, 2018) using deep learning-based predictions of infant brain age would benefit from advancing the prognostic validity of the brain age gap metric. For example, demonstrating associations between the metric and additional follow-up outcome metrics, such as executive function (Dai et al., 2021), would improve validity. Additionally, understanding the association between the metric and contemporaneous structural (e.g., body weight, brain structural MRI) and functional (e.g., sensory-evoked neural and behavioural responses, brain functional MRI) indices of development would be beneficial. For example, we recently showed that brain age predicted from sensory-evoked responses relates to electromyographic reflexes during preterm development (Zandvoort et al., 2024). Further investigations into these inter-relationships will be key to understanding the potential clinical value of brain age prediction models.

It is important to note that the focus of this manuscript was to provide an efficient approach for identifying abnormal brain maturation and to establish an association between brain age gap magnitude and longer-term neurodevelopmental outcomes. However, the causal role of brain age gaps in determining outcomes, as well as the potential environmental or genetic foundations for the brain age gap magnitudes, were not addressed in this study. There is increasing evidence that large brain age gaps may be a symptom of pre-existing conditions from birth (such as genetic factors or low birth weight), which has a lasting impact on the infant’s development presented through alterations in brain age trajectories (Vidal-Pineiro et al., 2021). While the underlying causal chains are only beginning to be explored in the literature, it is clear that the magnitudes of these brain age gaps are of biological and clinical interest. The ability to track and estimate brain age gaps with models such as the one presented here provides an easily implementable means to identify effects as soon as they manifest, potentially allowing for rapid clinical interventions.

It must be noted that the sample size used to train our age prediction model was very modest (111 recordings from 40 infants), while emerging best practice recommendations for prediction model development state that a minimum of several hundred observations are needed to be able to estimate meaningful prediction accuracies using cross-validation (Poldrack et al., 2019). In this study, we did not employ cross-validation to determine prediction accuracy in the training set (dataset 1), and in reporting our results for this training dataset, we do not wish to highlight estimates of prediction accuracy, other than to note that there was a minimal drop in accuracy observed between a model trained using 8-channel full duration EEG and our final model trained using 1-channel 20-minute EEG recordings. Instead, we entirely focus our assessment of model performance on two independent datasets from two clinical sites. The consistency between the prediction accuracies in these two independent datasets is noteworthy: MAE = 1.03 weeks for dataset 2 and MAE = 0.98 weeks for dataset 3. Furthermore, despite the modest sample sizes in both these test sets (148 recordings from 43 subjects and 73 recordings from 57 subjects), the accuracy of these estimates is reasonable and highly consistent: 95% CI in dataset 2 = [0.87, 1.28], and 95% CI in dataset 3 = [0.80, 1.19]. Undoubtedly, larger training and test sets would improve our model’s performance. However, it is clear that our limited sample size had little negative impact on model performance. This should be highly reassuring for researchers studying neonates in which data access limitations can be substantial.

In summary, in this study, we outline a deep learning approach for infant age prediction and follow-up BSID-II outcome risk stratification with reduced EEG data requirements relative to previous studies. In two independent held-out datasets, our model accurately predicts infant age and significantly distinguishes infants with normal outcomes from those with severely abnormal outcomes using a 1-channel bipolar montage setup and 20-minute recording duration. This objective and automated deep learning approach thus displays potential clinical utility for cot-side monitoring and use in neurological function assessment.

## Data availability statement

Due to ethical restrictions and the sensitive nature of these data, it is not possible to publicly share the supporting EEG data. For both test datasets 2 and 3, the PMA, predicted PMA, and group allocation for all recordings are provided, allowing the reproduction of all test dataset results. These data are available for download from Zenodo: https://doi.org/10.5281/zenodo.10993300.

## Code availability statement

The underlying code for the deep learning models, including the training, validation, and testing processes, is available for download using the following GitHub link: https://github.com/amirans65/brainagemodel. The underlying code for the test datasets analysis is available for download using the following Zenodo link: https://doi.org/10.5281/zenodo.10993300.

## Conflict of interest statement

None of the authors have potential conflicts of interest to be disclosed.

## CRediT authorship contribution statement

**Amir Ansari:** Methodology, Software, Validation, Formal analysis, Investigation, Writing – review & editing. **Kirubin Pillay:** Conceptualization, Methodology, Software, Validation, Formal analysis, Investigation, Data curation, Writing – review & editing. **Emad Arasteh:** Formal analysis, Writing – review & editing. **Anneleen Dereymaeker:** Investigation, Resources, Data curation, Writing – review & editing. **Gabriela Schmidt Mellado:** Visualization, Data curation, Writing – review & editing. **Katrien Jansen:** Investigation, Resources, Data curation, Writing – review & editing. **Anderson M. Winkler:** Formal analysis, Writing – review & editing. **Gunnar Naulaers:** Resources, Writing – review & editing, Supervision, Funding acquisition. **Aomesh Bhatt:** Writing – review & editing, Supervision. **Sabine Van Huffel:** Resources, Writing – review & editing, Supervision, Funding acquisition. **Caroline Hartley:** Data curation, Writing – review & editing, Supervision. **Maarten De Vos:** Conceptualization, Resources, Writing – review & editing, Supervision, Funding acquisition, Project administration. **Rebeccah Slater:** Resources, Writing – review & editing, Supervision, Funding acquisition, Project administration. **Luke Baxter:** Methodology, Formal analysis, Validation, Visualization, Writing – original draft, Project administration.
