# Supplementary material for: Resting state electroencephalographic brain activity in neonates can predict age and is indicative of neurodevelopmental outcome
Source: Clin Neurophysiol. 2024 Jul;163:226–35. doi: 10.1016/j.clinph.2024.05.002 (PMC11250083; doi:10.1016/j.clinph.2024.05.002)
Supplement: Supplementary Data 1 [file mmc1.pdf]

Supplementary Information

Resting state electroencephalographic brain activity in neonates can predict age and is indicative of neurodevelopmental outcome

Ansari et al.

S1. Exchangeability blocks

As outlined in the main text, we performed one-tailed significance testing, with a 5% significance level, performed using permutation testing via FSL's PALM: Freedman-Lane method with 10,000 permutations (Winkler et al., 2014). Due to multiple recordings per infant existing in both datasets, permutations were limited to appropriate exchangeability blocks, within which permutation is permissible. The hierarchical data structure, allowing visualisation of the exchangeability blocks, is displayed using tree diagrams as per the original methods paper (Winkler et al., 2015): Figure S1a displays the structure of dataset 2, and Figure S1b displays the structure of dataset 3.

a. b.

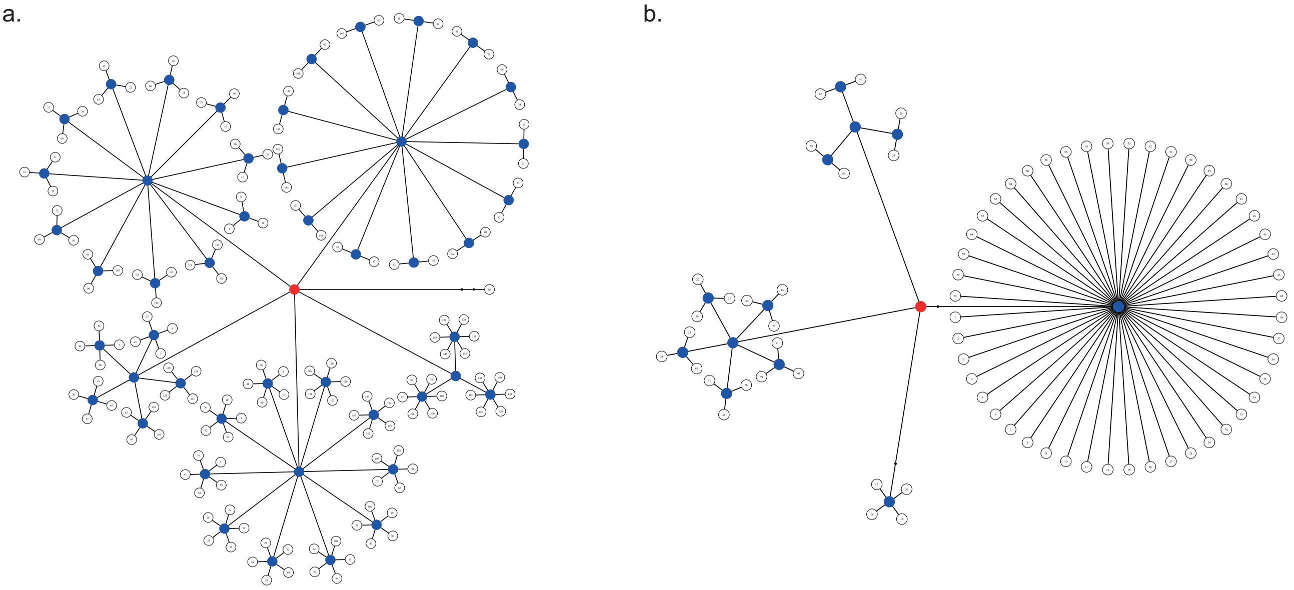

Figure S1: Tree diagrams depicting the hierarchical structure of both test datasets, defining exchangeability blocks for permutation testing. *Due to the differing number of recordings per subject, all recording age predictions are not fully independent or exchangeable within a dataset. These dependencies must be accounted for during statistical testing, and the tree diagrams display the relevant exchangeability blocks,* i.e.*, clusters of recordings in which exchangeability holds and permutations are permissible. Each white node represents a recording; each blue node connects clusters of recordings in which permutation is permissible. The red node represents paths connecting recordings through which permutation is not permissible. (a) Tree diagram for dataset 2. In this dataset (43 subjects, 148 recordings), there is one subject with one recording; 13 subjects with two recordings; 11 subjects with three recordings; five subjects with four recordings; 10 subjects with five recordings; and three subjects with six recordings. (b) Tree diagram for dataset 3. In this dataset (57 subjects, 73 recordings), there are 48 subjects with one recording; three subjects with two recordings; five subjects with three recordings; and one subject with four recordings.*

S2. Sensitivity tests

S2.1. Age is accurately predicted in two independent datasets using robust measures of performance

The trained model was applied to dataset 2, and performance was assessed using robust measures (median absolute error and robust R^2). The robust measures of performance were consistent with the measures (mean absolute error and R^2) presented in the main text: n = 43 subjects (148 recordings), robust R^2 = 0.90, median absolute error = 0.73 weeks, 95% CI = [0.59, 0.91], p=0.0001. Similarly, applying the model to dataset 3 and assessing performance using robust measures, the results were consistent with the measures presented in the main text: n = 57 subjects (73 recordings), robust R^2 = 0.78, MAE = 0.74 weeks, 95% CI = [0.59, 1.06], p=0.0001.

S2.2. Infant brain age gap, measured using median absolute error, contains clinically valuable prognostic information

We stratified the infants in dataset 2 based on their 9-month BSID-II outcomes: normal, mild abnormal, and severe abnormal. After correcting the prediction errors for PMA-association bias, we extracted a single brain age gap per infant by taking the median value across recordings (compared with the mean value that was taken in the main results). The infants with normal BSID outcomes had a mean brain age gap = 0.70 weeks (n = 22 infants, i.e., mean across infants of the median value across recordings), those with mild abnormal outcomes had a mean brain age gap = 0.87 weeks (n = 11 infants), and those with severe abnormal outcomes had a mean brain age gap = 1.15 weeks (n = 10 infants). These three groups are displayed using a Cumming estimation plot with the normal group as the shared control (Figure S2).

We performed pairwise comparisons of the mean brain age gaps among the three groups, adjusting for the number of recordings per subject and correcting p-values for multiple comparisons using a permutation testing approach. Among the three groups (n = 43 subjects), the severe abnormal BSID outcome group had a significantly larger mean brain age gap than the normal BSID outcome group: difference in mean brain age gap = 0.44 weeks, t-statistic = 2.90, p = 0.02, Cohen's D = 1.25. The other comparisons were non-significant. Severe vs mild BSID outcome group: difference in mean brain age gap = 0.26 weeks, t-statistic = 1.51, p = 0.31, Cohen's D = 0.74. Mild vs normal BSID outcome group: difference in mean brain age gap = 0.18 weeks, t-statistic = 1.23, p = 0.46, Cohen's D = 0.46.

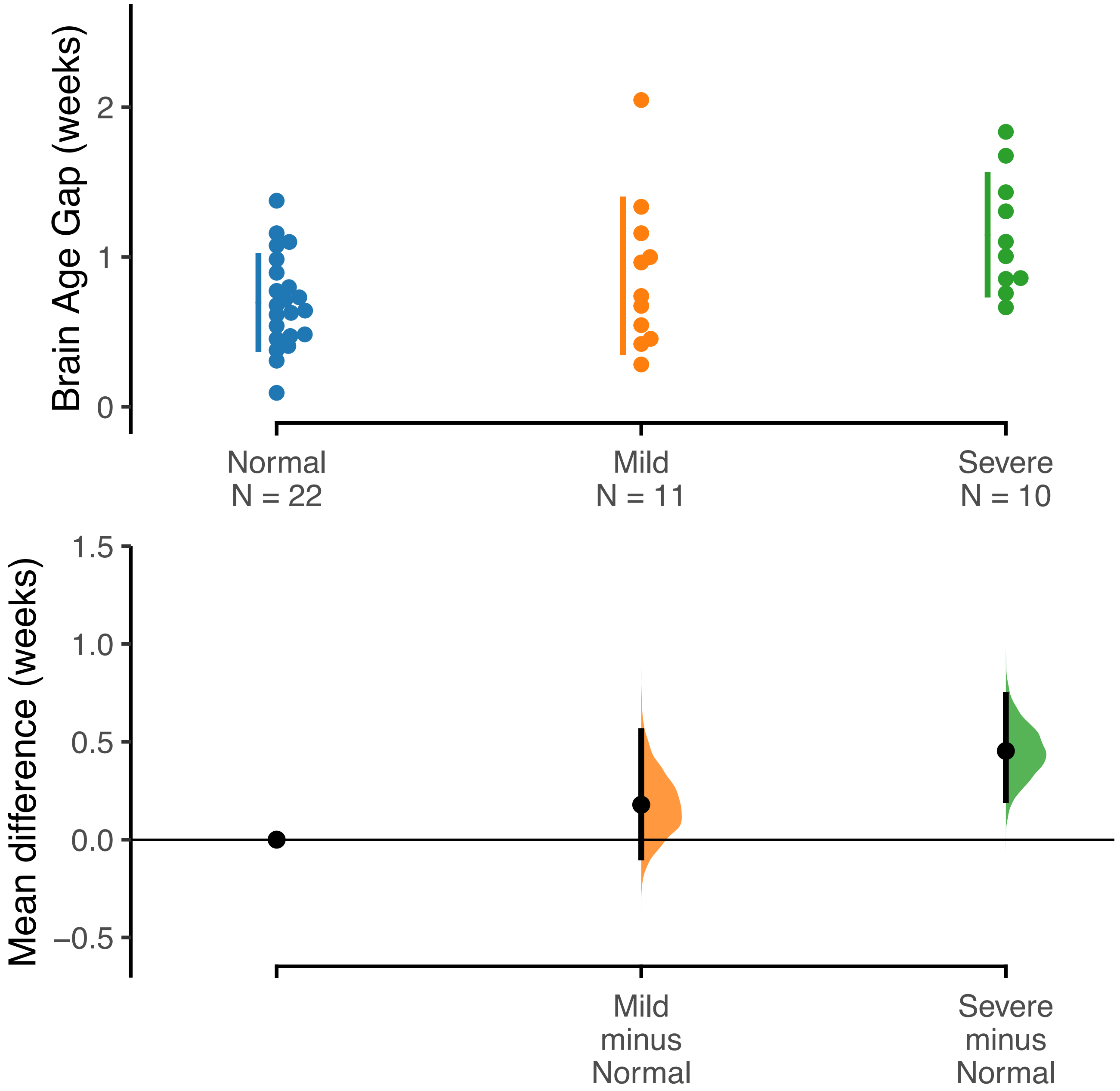

Figure S2. The brain age gap differed between groups of neonates stratified based on 9-month follow-up BSID-II outcomes. *The brain age gap results are displayed using a Cumming estimation plot. Top: In the swarm plots, each dot represents one subject, and subjects are grouped according to their BSID follow-up outcomes (normal is blue, mild abnormal is orange, and severe abnormal is green). The y-axis is the brain age gap magnitude: absolute value of prediction error with PMA association bias removed (residualised). Next to each swarm plot is a vertical line which is the ± standard deviation error bar. Bottom: The normal outcome group is used as a common control for the mild and severe abnormal groups. The solid circles represent the group mean minus the mean of the shared control, and the vertical black bars are the 95% confidence interval, determined using bootstrap resampling. The resampled distribution is also displayed. The severe outcome group had a significantly larger mean brain age gap (p-value = 0.02), assessed using two-sided t-tests, controlled for the number of recordings per subject, and p-values adjusted for multiple comparisons.*

S3. Supplementary references

Winkler, A.M., Ridgway, G.R., Webster, M.A., Smith, S.M., Nichols, T.E., 2014. Permutation inference for the general linear model. Neuroimage 92, 381–397. https://doi.org/10.1016/j.neuroimage.2014.01.060

Winkler, A.M., Webster, M.A., Vidaurre, D., Nichols, T.E., Smith, S.M., 2015. Multi-level block permutation. NeuroImage 123, 253–268. https://doi.org/10.1016/j.neuroimage.2015.05.092
